# Unexpected Malignancy: Squamous Cell Carcinoma Arising in an Ovarian Mature Cystic Teratoma Diagnosed Postoperatively

**DOI:** 10.7759/cureus.98775

**Published:** 2025-12-08

**Authors:** Chaimae Bekhakh, Anass Haloui, Mahmoud Aberkane, Nassira Karich, Amal Bennani

**Affiliations:** 1 Pathology Department, Mohammed VI University Hospital, Faculty of Medicine and Pharmacy, Mohammed First University, Oujda, MAR

**Keywords:** malignant transformation, mature cystic teratoma, ovarian germ cell tumor, ovary, squamous cell carcinoma

## Abstract

Although mature cystic teratomas (MCTs) of the ovary are typically benign, their rare malignant transformation (MT) into squamous cell carcinoma (SCC) represents a diagnostic and therapeutic challenge. Preoperative imaging frequently misleads clinicians by suggesting a benign lesion, and the malignant component is usually identified only after histopathological evaluation. Reporting such cases is valuable to raise awareness of this unusual entity and to emphasize the need for vigilance in the management of ovarian teratomas, particularly in women beyond reproductive age. We report the case of a 65-year-old woman in whom imaging suggested a benign teratoma, but histology revealed SCC, underlining the diagnostic challenge and the importance of awareness of this rare but clinically significant transformation.

## Introduction

Mature cystic teratoma (MCT), also known as a dermoid cyst, is the most common ovarian germ cell tumor, predominantly affecting women of reproductive age, with bilateral involvement reported in approximately 10-17% of cases [1]. Histologically, MCTs are composed of well-differentiated tissues derived from all three germ layers - ectoderm, mesoderm, and endoderm - and are usually asymptomatic, although some patients may experience abdominal pain or pelvic fullness due to the tumor’s mass effect [2]. Despite their benign nature, malignant transformation (MT) occurs in about 1-2% of cases, most frequently in older or postmenopausal women, with squamous cell carcinoma (SCC) accounting for 75-90% of these malignancies [3]. The preoperative diagnosis of MT remains challenging, as both clinical manifestations and imaging findings are often nonspecific, and malignancy is frequently identified incidentally during surgery or postoperative histopathological evaluation [4]. Given the poor prognosis of SCC arising in MCT and the absence of standardized therapeutic guidelines, early recognition through the integration of clinical, radiological, and histopathological findings is essential to guide timely surgical and adjuvant management [5].

The objective of this article is to report a case of SCC arising in an MCT, incidentally diagnosed during intraoperative pathological examination.

## Case presentation

A 65-year-old multiparous postmenopausal woman presented with severe, persistent abdominal pain and a palpable mass in the right lower quadrant (RLQ). She reported a six-month history of abdominal discomfort, which had worsened over the past three days. The pain was constant, non-positional, and non-radiating. She also experienced anorexia but denied fever or weight loss.

On physical examination, the abdomen was notably distended, with a large, firm, immobile, and tender mass measuring approximately 150 × 100 mm.

**Figure 1:**
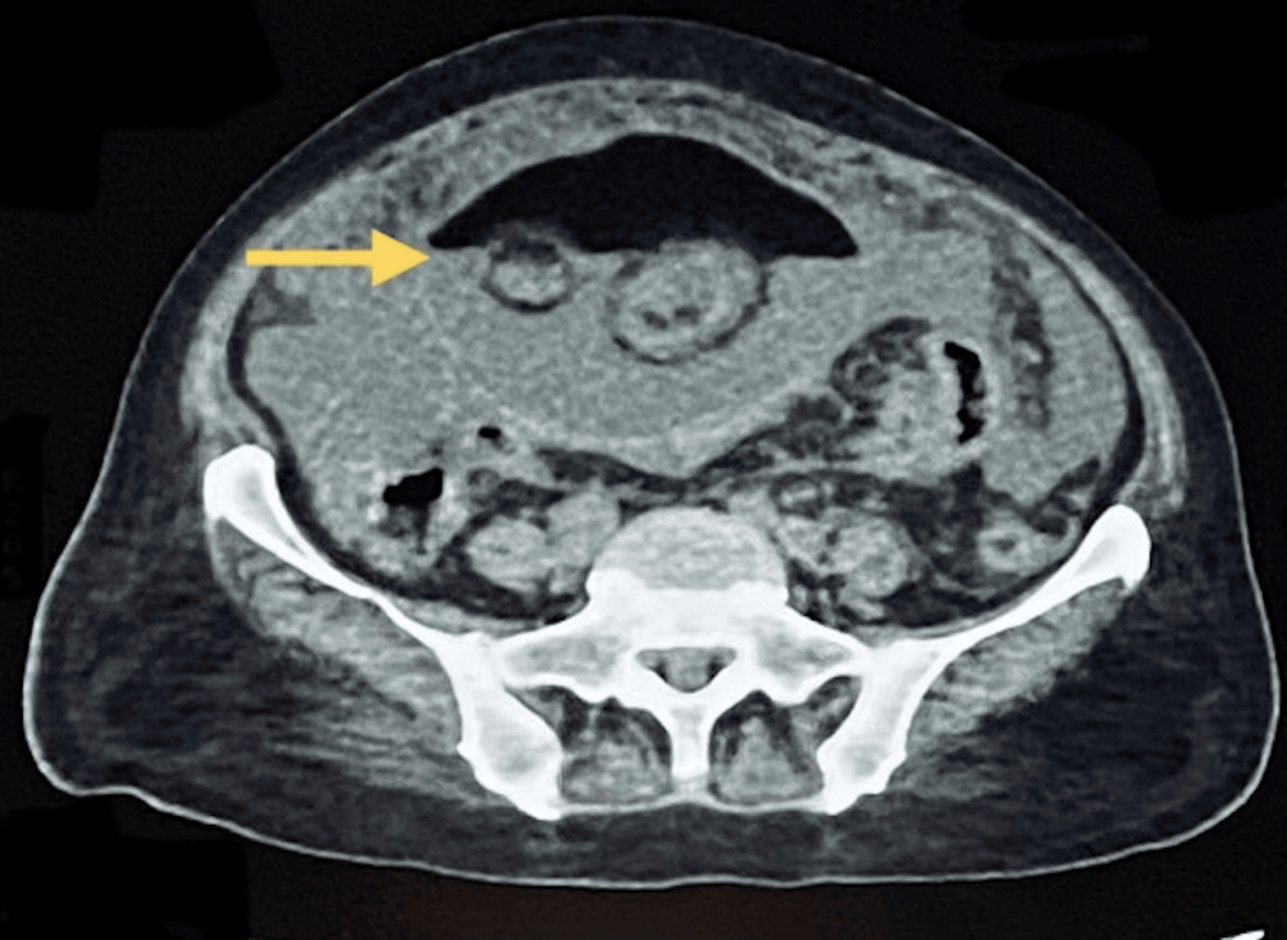
Large right ovarian mass measuring 160 × 125 × 86 mm, containing both fluid and fat components, suggestive of a teratoma (yellow arrow).

Computed tomography scan of the abdomen and pelvis revealed a large cystic mass in the right ovary measuring 160 × 125 × 86 mm. The mass exhibited heterogeneous content, including both fluid and fatty components, consistent with a mature cystic teratoma (dermoid cyst). There was no radiological evidence of extra-ovarian spread at the time of imaging (Figure 1).

To address the mass, the patient underwent an exploratory laparotomy performed by an oncosurgeon, including a right unilateral salpingo-oophorectomy and resection of the mass.

**Figure 2:**
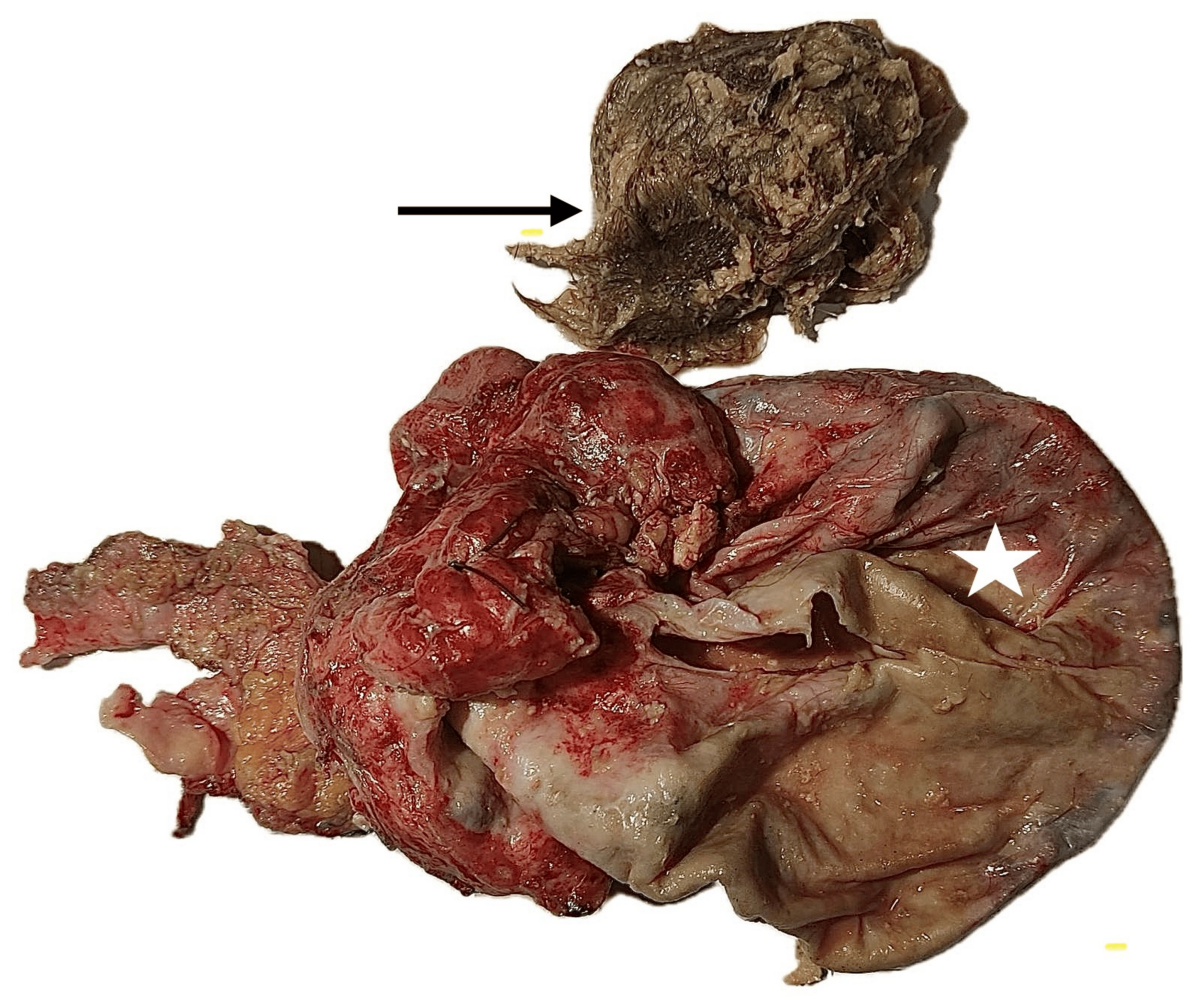
Ruptured right ovarian cyst (white star) containing hair and bone fragments (black arrow).

The histopathology report described an ovarian mass removed by right salpingo-oophorectomy, measuring 160 × 100 × 90 mm. On gross examination, the ovary was almost entirely replaced by a solid-cystic lesion. The cystic component accounted for approximately 50% of the mass and contained tufts of hair and a creamy-white sebaceous substance. The attached fallopian tube measured 50 mm in length (Figure 2).

**Figure 3:**
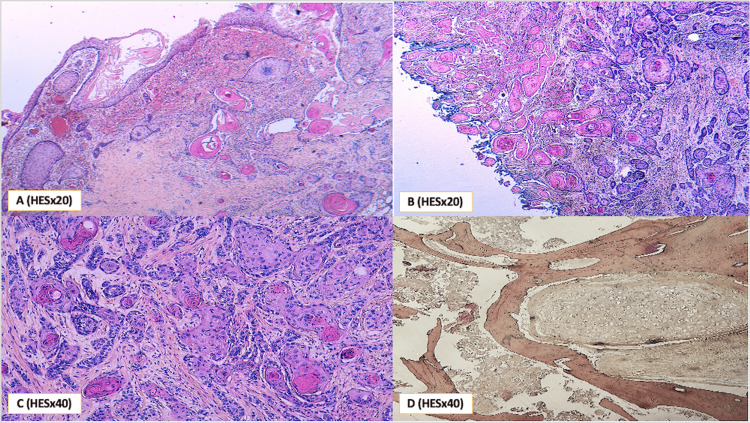
(A) Histological section showing surface carcinoma in situ with adjacent infiltrating nests. (B & C) Histological image showing a well-differentiated squamous cell carcinoma with extensive keratinization. (D) Mature lamellar bone and well-differentiated hyaline cartilage. HES: hematoxylin, eosin, and saffron

Histologically, the examined specimen revealed a cystic lesion containing a carcinomatous proliferation composed of solid sheets and trabecular structures. The tumor cells were large, with markedly atypical nuclei, prominent nucleoli, and numerous mitotic figures. The cytoplasm was abundant, eosinophilic, and displayed well-defined borders. Foci of maturation were observed in the form of keratin pearls. The lesion also contained elements of mature lamellar bone and well-differentiated hyaline cartilage, consistent with the presence of differentiated somatic tissues within the teratomatous background (Figure 3).

These findings were associated with extensive areas of necrosis and a frank capsular rupture. The tubal parenchyma appeared uninvolved, and no vascular emboli or perineural invasion were identified.

Overall, the diagnosis of MT of a mature cystic ovarian teratoma into SCC was established. The therapeutic decision was to initiate palliative chemotherapy. To date, the patient has undergone four chemotherapy sessions.

## Discussion

MT of an MCT of the ovary into SCC is a rare but well-recognized event, reported in approximately 1-2% of all MCTs. It predominantly affects peri- and postmenopausal women, whereas benign MCTs are most common in women of reproductive age. The mean age at diagnosis of SCC arising in MCT is between 50 and 60 years, and patient age is one of the most consistent predictors of malignant change [3,6].

Clinically, most patients present with abdominal pain, distension, or a palpable mass. Tumors are usually larger than benign dermoid cysts, with mean diameters ranging from 10 to 20 cm in malignant cases [4].

Radiologically, computed tomography (CT) and magnetic resonance imaging (MRI) may reveal a predominantly cystic mass with fatty and calcified components typical of MCT, but MT is suggested by irregular mural nodules, solid areas with contrast enhancement, or invasion of adjacent structures. However, these findings are not pathognomonic, and preoperative diagnosis remains challenging.

Serum tumor markers may aid in suspicion. Elevated SCC antigen (SCC-Ag) is detected in 30-60% of SCC-MCTs and correlates with tumor burden and recurrence risk. CA-125 may also be elevated but is less specific. Thus, a combined clinical, radiologic, and serologic evaluation is important for risk stratification [7].

Histologically, SCC arises from the squamous epithelium of the dermoid cyst lining. Malignant progression usually follows a stepwise sequence: squamous dysplasia → carcinoma in situ → invasive carcinoma. Microscopic features include nests, sheets, and keratin pearls of atypical squamous cells with high mitotic activity and stromal invasion. Immunohistochemistry typically shows strong positivity for p63 and CK5/6, while CK7 and CK20 are negative, confirming squamous differentiation [8].

A crucial aspect is the need for extensive sampling of large teratomas, as focal malignant areas may be missed if only limited sections are examined, particularly in predominantly cystic tumors. Therefore, pathologists recommend thorough sampling of any suspicious mural nodules or solid components [8,9].

Genomic studies have revealed recurrent alterations in TP53, PIK3CA, and CDKN2A, with frequent activation of the PI3K/AKT/mTOR pathway. Unlike cervical SCC, HPV DNA is rarely detected, suggesting that viral oncogenesis does not play a major role. This implies that intrinsic genetic instability of squamous epithelium within the dermoid drives carcinogenesis. Emerging data indicate that molecular characterization may identify potential therapeutic targets [10].

The mainstay of treatment is surgical staging, including hysterectomy, bilateral salpingo-oophorectomy, omentectomy, and lymphadenectomy, particularly in postmenopausal or advanced cases. Fertility-sparing surgery may be considered in strictly early-stage disease, although recurrence risk remains high. Adjuvant platinum-based chemotherapy (carboplatin-paclitaxel) is recommended beyond stage IA and has been shown to improve survival [1]. Radiotherapy offers no clear survival benefit and is not routinely advised. Targeted therapy with bevacizumab and immunotherapy with checkpoint inhibitors has shown promising results in isolated reports but remains investigational [1,11].

Although the pathophysiology of MT in MCT remains unclear, high-risk human papillomavirus (HPV) infection has been proposed as a potential causative factor through alterations in p53 and p16 expression [12]. In this case, relevant immunohistochemistry was not performed, highlighting the need for more extensive testing in such rare instances.

Prognosis is strongly stage-dependent. Five-year survival exceeds 80% in stage I disease but falls sharply to less than 20% in stage III-IV disease. Other adverse prognostic factors include tumor rupture, incomplete cytoreduction, elevated preoperative SCC-Ag, and capsular invasion [13].

Surveillance should include periodic imaging and serum markers (SCC-Ag and CA-125 if elevated preoperatively). Recurrences often occur within the first two years, emphasizing the importance of close follow-up during this period [13].

## Conclusions

SCC arising in a mature ovarian teratoma is rare but aggressive. Diagnosis relies on histopathological and immunohistochemical examination, while surgery remains the cornerstone of treatment. Early recurrence necessitates close follow-up. Reporting such cases enhances clinical understanding and informs future therapeutic strategies.
